# Is Belladona an Antidote to Opium?

**Published:** 1867-12

**Authors:** W. C. Bellamy

**Affiliations:** Columbus, Georgia


ATLANTA

Medical and Surgical Journal.

NEW SERIES.

VOL. VIII. DECEMBER, 1867. No. 10.

### ORIGINAL COMMUNICATIONS.

### ARTICLE I.

*Is Belladonna an Antidote to Opium?* By W. C. BELLAMY, Columbus, Georgia.

This is a question which has often been asked and answered both in the negative and the affirmative. It is, I believe, almost universally conceded that it is an antidote; but, being in itself such a deadly poison, its enemies claim that the danger in using it counterbalances whatever good effects that may arise from its use as an antidote. The writer of this article has happened to have quite an extended opportunity of forever setting at rest that point, at least, in his own mind. It has been his fortune, whether good or bad, to have met with perhaps more cases of poisoning than usually fall to the lot of the same amount of practice; and of several of the cases, he has made hasty sketches in his note book. In proof, therefore, of the decided value of Belladonna as an antidote to Opium, instead of any long-labored argument, he will proceed at once to give the history of several cases as taken from his note book. The first he will mention will be the case of a woman, one of the "fancy" sort, who, for some real or imaginary wrong, sought to end her miserable existence by self-destruction. May 13th, at 11 o'clock, was called in haste to see Julia P——, who, they said, had taken laudanum, and was dying. On arrival, found she had swallowed two oz. laudanum about half an hour previously. She was perfectly insensible; suffering with the most violent convulsions, pupils very much contracted, raving, rolling, tossing, tearing her head, foaming at the mouth, groaning, grunting, screaming, striking at the bed and every one who approached her, trying to tear her flesh, and throwing herself about with such almost superhuman strength, that it required two stout women and a strong man to hold her on the bed. Her skin, however, felt natural, and her pulse almost so, being only a little excited above the normal standard. I ordered the attendants to gag her, she being too insensible to be managed otherwise, and made her swallow, immediately, about twenty grains of sulphate of zinc, and one or two of tartar emetic, in a little water. Waiting a few minutes, during which the convulsions continued and no emesis, I repeated the sulphate of zinc, after which, in a few minutes, she vomited freely. I discovered in the egesta a large quantity of laudanum, from the smell, and some whisky. She continued vomiting sometime. The convulsions would occasionally subside, but would return almost instantly; and, I think, she must have had at least two hundred convulsions before they ceased entirely. After she had vomited till she could vomit no more, I ordered a teaspoonful of the tincture belladonna every half hour, then every hour, watching the effect myself, not leaving this to her nurses. After awhile, I had the satisfaction of seeing the pupils begin to dilate, and the spasms to abate, both in frequency and violence, and about 4 o'clock in the afternoon, the spasms had almost entirely ceased, and she was disposed to sleep. I ordered then 30 drops of tincture belladonna every hour, and frequent draughts of a decoction of green coffee, and for her to be kept awake by force all night, and left her. I returned next morning, found the spasms entirely ceased, the pupils natural, and the patient out of danger. I ordered them to stop the belladonna; to give her coffee and a good brandy toddy, and allow her to sleep, which she did from then till the next morning, when she awoke and convalesced rapidly, and is now well.

The next case was that of a mother, by mistake, giving to her babe, about nine months old, several teaspoonsful (she did not know how many) of laudanum. It presented pretty much the same symptoms as the foregoing patient did, and, on arrival, I gave it ipecac and warm salt water, and soon produced free emesis. The child was perfectly insensible, as "limber as a rag," to use a common expression, and the pupils contracted to a mere point. As soon as free emesis was effected, and the child had vomited till it could vomit no more, I ordered six drops of tincture belladonna in a little syrup of orange peeling every half hour, till a perceptible effect was visible upon the pupil. I gradually decreased the dose, and by the next day the child was well.

The third case was that of the child of a dear friend of mine, who, by mistake, had been given about a grain of morphine instead of quinine. The mistake was not discovered until the strange appearance gave evidence that something was wrong, or until the mother thought the child was dying. Several physicians, seven in all, I believe, were called in; and when I saw the child, it was in a perfect state of collapse. The physician first there worked very faithfully with the child, not being willing to trust to an emetic to empty the stomach, and, not having a stomach pump, one of the physicians inserted a long G. E. catheter into the stomach and extracted the contents by sucking them out with his mouth. The child was stripped, placed in a bath and rubbed and slapped with cold wet cloths. Milk, decoction of coffee, castor oil, spirits turpentine, and various other articles, passed into the stomach through this G. E. catheter, and he was kept awake by being jumped, jolted, slapped on his naked skin with cold wet cloths, etc.; but there appeared no change in his symptoms during the whole of the afternoon. Most of the physicians in attendance were my seniors, and objected to my recommendation of the belladonna. All, however, by 9 o'clock, except myself and one other, had left. I then begged my friend to let us try the effects of the belladonna, as we saw his child must die unless there was some change. He and the only other physician remaining present consented. We, therefore, gave the child seven drops of the tincture, and in a half hour thought we saw some change in the pupil. We repeated the dose, and in another half hour saw a decided change; in another half hour, after having given him this time six drops, we saw that he was rapidly reviving under its influence; and, after having given him several more doses, diminishing the dose one drop each time, we had the satisfaction of feeling that he was out of danger, and by morning, on the following day, we considered him sufficiently so to allow him to sleep as much as he pleased. He enjoyed, after it all, a refreshing sleep, and, when he awoke, found himself quite well, with the exception of fatigue and the soreness from jumping, jolting and slapping to keep him awake.

It may be remarked here, that the child had had intermittent fever, and, by mistake, had taken the morphine for quinine, and that, after having recovered from the poisonous effects of the morphine, it was cured of the chill and fever.

I merely give these cases as direct proof of the inestimable value of belladonna as an antidote to opium, and to show, at the same time, what a quantity of it can be borne by one poisoned by opium. And this, I hope, will overthrow what I consider a false idea, that the "remedy is worse than the disease," and establish the fact that, in cases of poisoning by opium, we have a safe, quick and reliable antidote in belladonna. I may also remark here, too, that I have known it equally as effective, given by subcutaneous injections, if, from any cause, it be impracticable to introduce it into the stomach.

---

